# Adaptable Functionality of Transcriptional Feedback in Bacterial Two-Component Systems

**DOI:** 10.1371/journal.pcbi.1000676

**Published:** 2010-02-12

**Authors:** J. Christian J. Ray, Oleg A. Igoshin

**Affiliations:** Department of Bioengineering, Rice University, Houston, Texas, United States of America; University of Pennsylvania, United States of America

## Abstract

A widespread mechanism of bacterial signaling occurs through two-component systems, comprised of a sensor histidine kinase (SHK) and a transcriptional response regulator (RR). The SHK activates RR by phosphorylation. The most common two-component system structure involves expression from a single operon, the transcription of which is activated by its own phosphorylated RR. The role of this feedback is poorly understood, but it has been associated with an overshooting kinetic response and with fast recovery of previous interrupted signaling events in different systems. Mathematical models show that overshoot is only attainable with negative feedback that also improves response time. Our models also predict that fast recovery of previous interrupted signaling depends on high accumulation of SHK and RR, which is more likely in a positive feedback regime. We use Monte Carlo sampling of the parameter space to explore the range of attainable model behaviors. The model predicts that the effective feedback sign can change from negative to positive depending on the signal level. Variations in two-component system architectures and parameters may therefore have evolved to optimize responses in different bacterial lifestyles. We propose a conceptual model where low signal conditions result in a responsive system with effectively negative feedback while high signal conditions with positive feedback favor persistence of system output.

## Author Summary

Bacteria have evolved various mechanisms for surviving unpredictable changes and stresses in the environment, such as nutrient limitation. One common survival mechanism is the two-component system, where a sensor protein responds to a particular type of stress by activating a regulator in the cell. These regulators can in turn activate genes that produce proteins for stress-appropriate responses. The activated regulator often positively regulates transcription of its own operon containing the sensor and regulator genes leading to a feedback loop. This is interesting, because positive feedback is usually associated with a slower response time than negative feedback and therefore negative feedback would often be selected for by evolution. Here we analyze a mathematical model to study the interplay of this feedback and postranslational mechanisms regulating two-component system signaling. We found that modulation of regulator activity by its operon partner can lead to overall negative feedback to result from autoactivation. This happens if (1) the sensor can both activate and deactivate the regulator, and (2) there is some reaction resulting in regulator activation independently of its cognate sensor. As a result our model predicts that two-component systems may be capable of flexibly switching between positive and negative feedback depending on different circumstances, allowing for appropriate responses in a variety of conditions.

## Introduction

Unpredictably changing environments necessitate appropriate responses for successful survival by bacteria. Bacterial two-component system (TCS) signaling shifts transcriptional programs in response to a variety of external cues affecting bacterial growth such as nutrient availability, osmolarity, redox state, temperature, and concentrations of other important extracellular molecules [1]. The basic TCS core structure includes a sensor histidine kinase (SHK) and response regulator (RR). SHK-modulated phosphorylation of RR results in activation that frequently induces a transcriptional program.

Environmental signals are routed through conformational changes in SHK homodimers, which may participate in up to three biochemical processes. First, each subunit hydrolyzes ATP to trans-phosphorylate a His residue in the other subunit. Second, the phosphorylated SHK subunit transfers its phosphate to an Asp residue on unphosphorylated RR bound to the SHK. Third, in bifunctional TCSs, unphosphorylated SHK can catalyze dephosphorylation of RRP with release of inorganic phosphate. Modulation of phosphatase and/or kinase activities of SHK may therefore induce system responses by shifting the dynamic equilibrium between active (phosphorylated) and inactive (unphosphorylated) forms of RR. Interactions between the RR and exogenous phosphodonors (e.g. cross-talk with non-cognate TCSs or small molecule phosphodonors) may also contribute to TCS activation.

**Figure 1:**
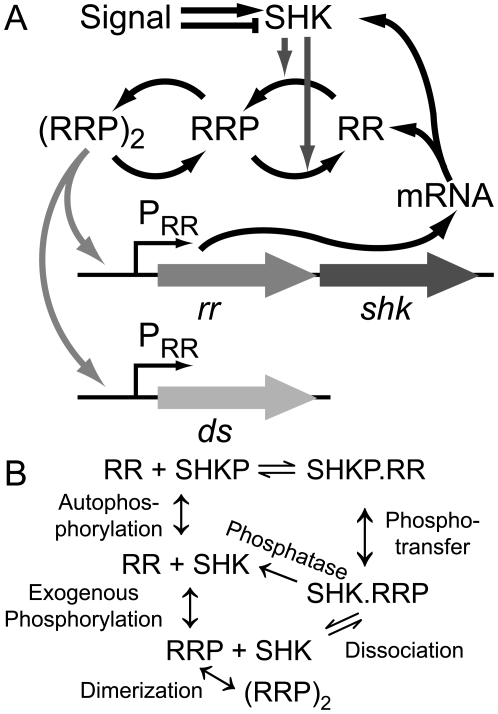
Schematics of two-component system production and regulation. A. A single operon expresses response regulator (RR) and sensor histidine kinase (SHK). Bifunctional SHK modulates RR phosphorylation. Dimerized phosphorylated RR regulates its own operon as well as downstream proteins. B. Post-transcriptional biochemical mechanisms. ↕ denotes reactions modeled as monomolecular; 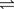 and → denote reversible and irreversible bimolecular reactions, respectively. Reaction mechanisms include protein-protein binding and unbinding and phosphorylation (including from exogenous sources for free RR). Each complex is subject to growth dilution; mRNA undergoes degradation (not depicted for clarity). Tables 1 and S1 present a quantitative formulation of the model.

Activated RR may induce expression of multiple operons. This regulon often contains the operon encoding the RR and SHK. Such autoregulation (Figure 1A) is observed in PhoPQ [2], PhoBR [3], VanRS [4], CpxRA [5], CusRS [6], and many other model systems [7]. The physiological significance of this feedback loop is not completely understood.

Previous genomic studies in *E. coli* have shown widespread positive and negative transcriptional autoregulation [8]–[10]. These studies indicate several *E. coli* TCSs as examples of positive feedback loops. However, we suggest that the effective sign of feedback in TCSs may depend on the biochemical interactions of the autoregulated proteins. Induction of bifunctional SHK in the same operon as RR may affect the phosphorylation equilibrium, and therefore have either positive or negative effects on the amount of transcriptionally active RR. That is, the signal increases RR phosphorylation, but resultant increases in gene expression may in turn positively or negatively change the amount of phosphorylated RR amplifying or attenuating the original signal. The resulting sign of feedback can be related to the transient dynamics of TCS activation: overshoot kinetics often result from underdamped negative feedback [11], and have been observed in one TCS [12]. The attainment of such overshoot has important implications on the kinetics: it transiently speeds expression of genes in the regulon (e.g. [13] and below) and is necessary for virulence of *Salmonella enterica* serovar Typhimurium (hereafter, *Salmonella*) in mice [12].

We used a mathematical model of a generic TCS to demonstrate that post-translational kinetics of SHK-RR interactions can determine the effective sign of feedback. The model explains disparate results relating to transcriptional autoregulation in TCSs including the “learning” effect [3] and feedback-induced surge [12]. Moreover, we show that some systems may display both effectively negative and positive feedback at different signaling levels. The effective feedback sign is determined by kinetic parameters of TCSs, with positive and negative feedback allowing distinct functional advantages in different circumstances. Therefore, differences in post-transcriptional kinetics may have arisen from selective pressure for feedback based on bacterial lifestyle. The accessibility of either type of feedback in the same system also raises the possibility of tuning between “responsive” and “persistent” signaling modes in a single TCS.

## Results

### Dynamical model of a two-component system with transcriptional feedback

To determine the role of transcriptional feedback in TCSs, we constructed an ordinary differential equation model extending previous work [14],[15]. The resulting model is schematically represented in Figure 1A; reaction mechanisms are presented in Figure 1B. The full set of reactions is listed in Table 1.

**Table 1:** Two-component system model with transcriptional feedback.

Reaction Number	Reaction	Rate/Flux
1	→ TCS mRNA (RRP_2_)	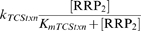
2	→ DS mRNA (RRP_2_)	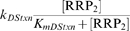
3	→ TCS mRNA	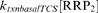
4	→ DS mRNA	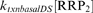
5	mRNAs → Ø	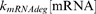
6	→ SHK (TCS mRNA)	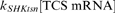
7	→ RR (TCS mRNA)	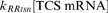
8	→ DS (DS mRNA)	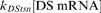
9	Proteins → Ø	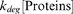
10	SHK → SHKP	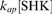
11	SHKP → SHK	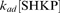
12	SHK + RRP → SHK.RRP	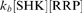
13	SHKP + RR → SHKP.RR	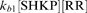
14	SHKP.RR → SHK.RRP	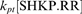
15	SHK.RRP → SHKP.RR	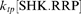
16	SHK.RRP → SHK + RRP	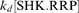
17	SHKP.RR → SHKP + RR	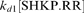
18	SHK.RRP → SHK + RR	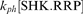
19	RR → RRP	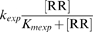
20	RRP → RR	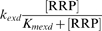
21	2 RRP → RRP_2_	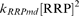
22	RRP_2_ → 2 RRP	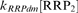

A signal may modulate the rate of kinase (*k_ap_* in Reaction 10) or phosphatase (*k_ph_* in Reaction 18) activity of SHKs [16]. We usually take the kinase activity to be constitutive as with *Salmonella* PhoPQ [17], but demonstrate the generality of our results to either type of modulation below.

A critical component of this model is the existence of an SHK-independent flux of RR phosphorylation and dephosphorylation, arising from small molecule phosphodonors, autodephosphorylation, or crosstalk with other TCSs [1], [18]–[20]. We assume a Michaelis-Menten form for these fluxes that is biologically consistent with the crosstalk mechanism: 
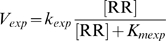
 (Reaction 19); 
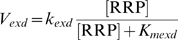
 (Reaction 20). The possibility of small molecule phosphodonors as the source may result in linear phosphorylation/dephosphorylation kinetics, resulting in qualitatively similar results: 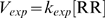;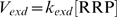. For brevity we present only the results using the Michaelis-Menten form.

### Sign of feedback depends on posttranslational interactions of two-component systems

To study TCS induction dynamics with the model, we chose a Monte Carlo parameter sampling approach because no general analytical solution of transient response is possible. Signal level was determined by parameter *k_ph_*, held at 10/s for the resting steady state and changed to 0.1/s to activate at *t* = 0 min. The effective sign of feedback in the model is measured by open-loop gain 
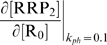
 at the activated steady state. That is, we take a no-feedback, or open loop, form of the model in which the operon is activated by putative exogenous activator R_0_. The gain measures how changes in R_0_ concentration affect the concentration of transcriptionally active response regulator RRP_2_ (at a concentration of R_0_ equal to the activated steady-state RRP_2_ concentration). Intuitively, the feedback increases total RR and SHK concentrations. However, increases in these concentrations may have a positive or negative effect on the phosphorylated fraction of RR. These conditions respectively correspond to effectively positive and negative feedback. The open-loop gain is notably different from the steady-state signal-response gain of the system. In all cases considered, system response (level of activated RR) increased with increased signal (decreased *k_ph_*). Nevertheless, the sign of open-loop gain can be either positive and negative for the cases in which transcriptional feedback respectively amplifies or attenuates RRP concentration.

**Figure 2:**
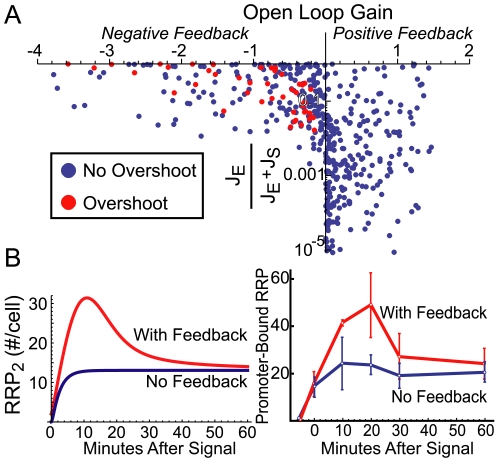
Conditions for negative feedback and overshoot in a two-component system model. A. Monte Carlo parameter sampling shows that both positive and negative feedback are attainable with the model. Overshoot kinetics only occur when the feedback sign is negative. The *y* axis is fraction of contribution of J_E_, the flux of exogenous response regulator phosphorylation, relative to the total phosphorylation flux that also includes J_S_, the flux of sensor histidine kinase-mediated phosphorylation. Negative feedback cases all have a proportion of exogenous flux above ∼0.001 (i.e. 0.1%). “Overshoot” cases denote a peak phosphorylated response regulator dimer (RRP)_2_ concentration greater than 50% above the activated steady state. Open-loop gain is calculated as response of the open-loop system to perturbation of an exogenous regulator (R_0_) at the activated steady state (*k_ph_* = 0.1). B. Comparison of simulated overshoot kinetics and experimental measurement of *mgtA*-bound RRP (phospho-PhoP) in *Salmonella* (adapted from 11). The “No Feedback” case takes a constitutive expression level equal to the activated steady state of the “With Feedback” case in the simulations. Simulations used the reference parameter set in Table S1.

Parameter sampling returns both negative and positive open-loop gains corresponding to overall negative and positive sign of feedback (Figure 2A). In each case with a negative loop, some fraction of RR phosphorylation results from non-cognate sources (*i.e.*, J_E_/(J_E_+J_S_)>10^−3^ where J_E_ is flux through Reaction 19 and J_S_ is flux through reaction 16 in Table 1). An exogenous RR phosphorylation flux was the only identified mechanism to produce a negative feedback loop in a more generalized TCS model as well (Text S1). More extensive sampling of a simplified model confirms these results (Text S2; Figure S3).

### Dynamical properties of the system correlate with feedback sign

A common dynamical characteristic of negative feedback is overshoot kinetics, which have been shown to occur in PhoPQ of *Salmonella* [12]. A subset of randomly generated parameters results in the prediction of feedback-induced overshoot in concentrations of RRP and mRNA of genes under its control (Figure 2A, red circles). Notably, the effective sign of feedback is negative for all of them. Figure 2B shows a sample time-course selected to resemble *Salmonella* PhoPQ compared with experimental results of downstream promoter binding from [12]. We used a genetic algorithm to select parameter sets for systems resembling PhoPQ; one of the representative sets is used as a default example throughout the text (Table S1; Figure 2B). Briefly, we selected for significant feedback-modulated overshoot in RRP and significant increases in both total RR and transcriptionally active RRP_2_ at the activated steady state. Figure S1 shows further dynamical characteristics of the reference system.

**Figure 3:**
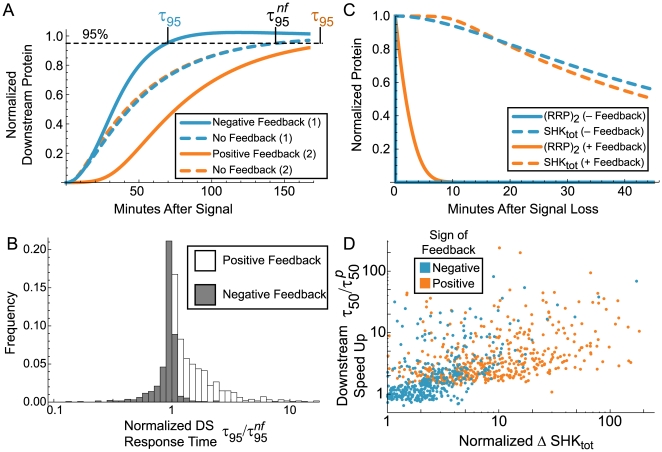
Dynamic characteristics of positive and negative feedback in two-component systems. A. Two example parameter sets from Monte Carlo sampling show that a downstream protein typically responds faster under negative feedback than positive. The two cases were selected to have similar induction kinetics in the model lacking feedback. τ_95_: time to attain 95% of the activated steady state from the resting state. B. Histogram of response time ratios of sampled parameter sets shows the relationship between feedback and response time for many parameter sets generated with Monte Carlo sampling. 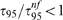 implies that feedback improves response time; 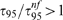 implies that feedback is detrimental to a fast response. C. Deactivation kinetics in example cases show two time scales in both positive and negative feedback. D. Timing of RRP recovery after signal interruption correlates with normalized (percent) accumulation of SHK protein concentration (ΔSHK_tot_). The scatter plot shows the relationship between ΔSHK_tot_ and change in response time. Response time 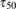 is the time to attain 50% of the activated steady state RRP_tot_ from the resting state; 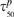 is the response time following a previous stimulus and 45 minute interruption. *y*-axis values greater than 1 denote faster response after interrupted prior stimulus. The Spearman rank correlation is ∼0.625 (*p*∼0 with double machine precision). See Methods for parameters used.

In simple inducible genetic systems with autoregulation, negative feedback is associated with faster attainment of a given steady state as compared to positive feedback [21],[22]. In the TCS model, we compared the response (here, downstream (DS) protein accumulation) between a feedback-regulated model and an open-loop model where constitutive gene expression in the open-loop form equals expression at the activated steady state of the closed loop model (Figure 3A–B). The results are consistent with negative open-loop gain speeding responses and positive open-loop gain slowing responses compared to the case with no feedback going to the same steady state. The advantage of negative open-loop gain is especially pronounced with feedback-induced overshoot. This result confirms that the effective feedback sign as measured by open-loop gain corresponds to the directly observable feedback sign in simpler systems. Text S3 and Figure S4 discuss the small number of cases in Figure 2B that appear to contradict this rule.

Another kinetic effect of feedback in TCSs is “learning” exhibited in *E. coli* PhoBR, where responses to a second stimulus after a transient signal interruption are faster than the response to the first signal [3]. To explore the “learning” effect, we determined the response time of downstream protein accumulation after a 45 minute signal interruption (Figure 3C–D) for sampled parameter sets and compared this response with the one following the initial signal. The length of the signal interregnum was chosen to reflect prior experiments [3]. The 45 minute interregnum, longer than the timescale of dephosphorylation (seconds to minutes), but shorter than the timescale for protein dilution to near basal levels (hours), is biologically reasonable (Figure 3C simulates this in the TCS model). The results predict that the “learning” effect can be observed in both positive and negative feedback, but is more pronounced for positive feedback. Improvements in downstream response depend on accumulation of SHK and RR protein concentrations during the first activation (Figure 3D; Spearman's rank correlation ∼0.625; *p*∼0 with double machine precision). High induction capacity attainable by most positive feedback cases amplifies the “learning” effect; it does not arise from bistability or slow response times usually associated with positive feedback [21],[23]. In fact, the phosphorylated fraction of RR responds quickly to changes in signaling; it is the accumulation in protein level that causes this effect (Figure 3C). Therefore, our results correlate TCSs functioning in positive feedback mode with faster recovery following transient signal fluctuations.

### Effective feedback sign depends on signal level

**Figure 4:**
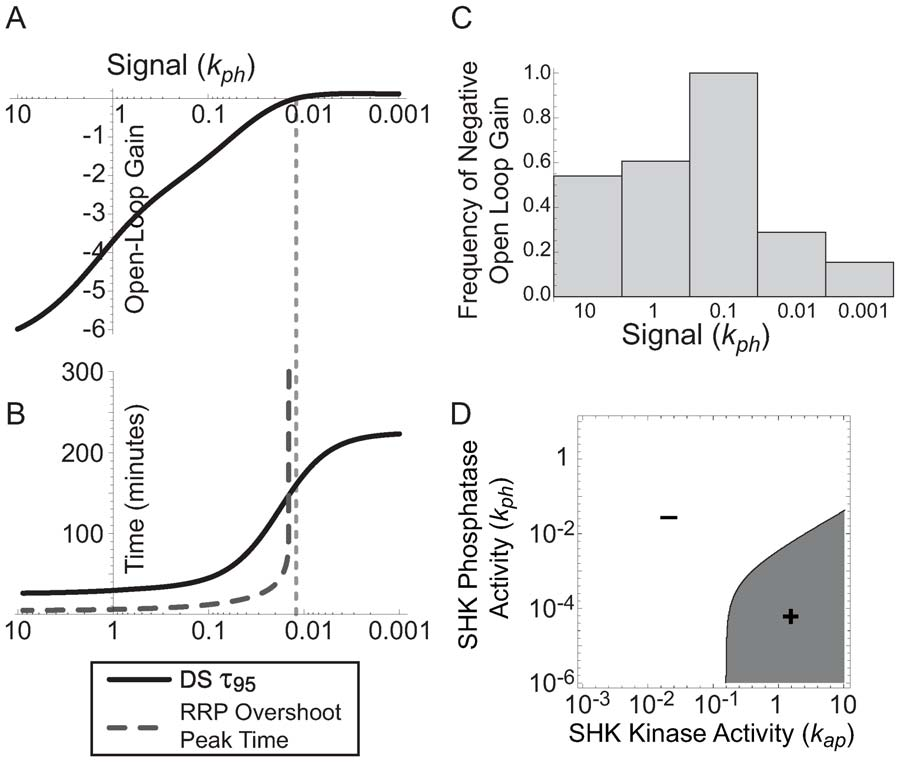
The effective sign of transcriptional feedback in two-component systems can reverse depending on signal strength. A. For the sample set of parameters (Table S1) feedback is negative at low activation signal and becomes positive for sufficiently high signal (low *k_ph_*). B. Dynamic properties (response time and overshoot peak time for initially inactivated system, *k_ph_* = 10) follow the effective sign of feedback at different signal levels; parameters the same as in A. Dashed line indicates the point of sign switching; at this point overshoot time approaches infinity. C. Monte Carlo sampling demonstrates that systems selected to have effectively negative feedback at a given signal level (*k_ph_* = 0.1) robustly switch to positive feedback at other signal levels. D. Sign of open-loop gain can switch while varying both kinase (*k_ap_*) and phosphatase (*k_ph_*) activities of the sensor histidine kinase; the rest of parameters the same as in (A). Gray: positive open-loop gain. White: negative open-loop gain.

The sign of feedback is a function of parameters in the model presented here; it is therefore conceivable that specific parameter perturbations could change the strength or sign of feedback. We found a large number of cases in which effective feedback signs change as a function of input signal strength (parameter *k_ph_*). The majority of Monte Carlo parameter samples that predict negative feedback for one signaling level exhibit positive feedback for a different signal level (Figure 4A).

If wild-type TCSs are indeed capable of changing feedback sign, the dynamic performance of the system may be capable of meeting different functional criteria depending on the signal level. To determine if feedback sign changes performance in TCSs, we compared, at various signal levels (*k_ph_*), open-loop gain and response times of downstream protein induction from the basal state (*k_ph_* = 10/s) using the default parameter set (Figure 4B–C). Response times correspond directly to the effective sign of feedback at each activation level: negative feedback imparts faster response. Furthermore, peak overshoot time approaches infinity as the signal level approaches the positive feedback region (Figure 4C dashed line). Therefore the TCS model predicts that adapting the feedback sign based on transient signal intensity permits TCSs to meet different functional criteria depending on the context.

Unmasking of positive feedback for the high signal region of the dose-response curve may explain the strong effect of feedback in *E. coli* TCS PhoPQ that only occurs for extreme signaling conditions [24]. We explore other aspects of the *E. coli* PhoPQ dose response in Text S4 and Figure S5.

TCSs may be subject to modulation of SHK kinase activity (parameter *k_ap_* in this model) or both kinase and phosphatase activity (*k_ap_* and *k_ph_*). We scanned the 2-dimensional *k_ap_* × *k_ph_* signal space and found that modulation of either activity may result in feedback tuning (Figure 4D).

### Synergies and trade-offs in transcriptional feedback interactions

**Figure 5:**
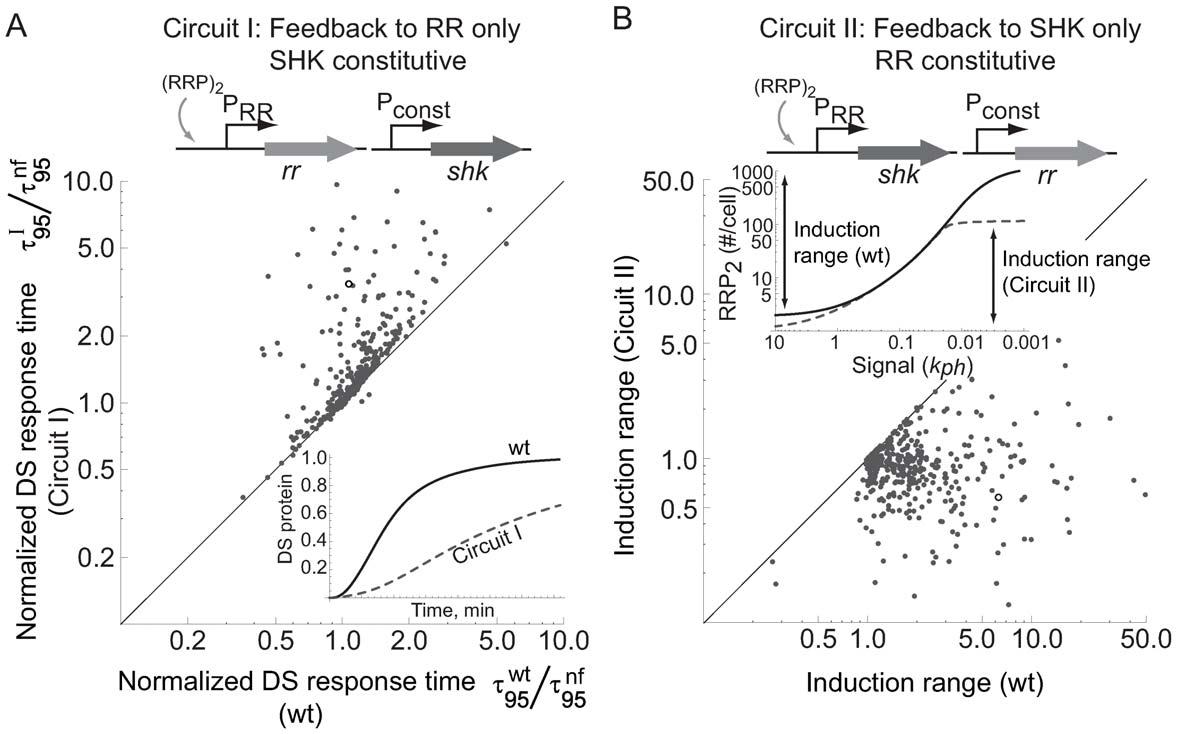
Tradeoff and synergy in two-component system feedback interactions. A. Dynamic response of downstream (DS) protein expression for Circuit I (feedback to RR alone) is slower than the wild-type architecture. 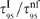 denotes time to attain 95% of the activated steady state of downstream protein (with *k_ph_* = 0.1) in the system with constitutive expression of the *shk* gene, normalized to the same system with no feedback. 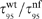 is the same ratio for wild-type system with both SHK and RR under feedback regulation. B. Steady state dose-response shows reduced induction range for 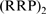 in Circuit II (feedback to SHK alone) as compared to the wild-type (single operon) case. Induction range denotes steady-state difference between phospho-RR dimer with *k_ph_* = 0 to find the highest, saturated level, and with *k_ph_* = 10 (basal expression) in the wild-type (wt) case and the case with constitutive expression of the *rr* gene, again normalized to the same system with no feedback. Insets show kinetics and dose-response of reference parameter set (Table 2; open circles on scatter plots). Constitutive gene expression is set to equal feedback-regulated expression at high signal, *k_ph_* = 0.1.

Autoregulation by RRP proportionally affects concentrations of both RR and SHK in the genetic architecture assumed here – both genes co-expressed from a single operon (Figure 1). Is there a functional rationale for why feedback affects both genes in many TCSs, as opposed to autoregulation of only RR or SHK? To address this question, we formulated models where expression of one of the TCS proteins, either RR or SHK, is constitutive (outside of the TCS regulon) whereas the other is regulated by RRP (Circuits I and II, Figure 5). The constitutive rate of production is set equal to the production rate in the wild-type system with the signal parameter *k_ph_* = 0.1/s. This level was chosen to represent a production rate that avoids saturation effects of excessively high or low expression.

When RRP only regulates the RR gene (Circuit I), the response time is typically slow (Figure 5A). When RRP only regulates SHK (Circuit II) the induction range (defined as the difference between high and low signal limits, normalized to the case without feedback) is typically smaller (Figure 5B). This suggests that the wild-type case, with both RR and SHK feedback-regulated from a single operon, exhibits a trade-off, balancing fast response and high induction range. At higher signal levels, the wild-type system exhibits a synergistic effect where the linked genes can attain higher signal output than with feedback to RR alone (Figure 5B).

## Discussion

TCSs are responsible for diverse, specialized, large-scale reprogramming of bacterial transcription in response to many possible signals. Nevertheless, while orthologous TCSs in different species often have drastically different regulons [25], core TCS architectures are remarkably similar. Some TCSs are autoregulated, where transcriptionally active RR induces expression of the TCS operon. This feedback may in itself have diverse effects on the system, including overshoot kinetics [12] and “learning” to respond faster after a previous stimulus [3]. In the interest of generality we focused on a commonly occurring TCS architecture, with linked genes that are autoregulated. Others not considered here may have important functional consequences (e.g. bistability emerging from autoregulation in the *B. subtilis* DegS-DegU system [26]).

### Feedback sign results from biochemical mechanisms

Despite the fact that autoregulation is almost uniformly positive in TCSs (with the exception of TorRS in *E. coli* [27]), we have shown that the effective feedback sign may be either positive or negative, depending on biochemical characteristics of the system. Why is attainment of different feedback signs physiologically relevant? In most biological systems, negative feedback often reduces noise and speeds responses in well-controlled comparisons [21],[28],[29] while positive feedback leads to phenotypic heterogeneity and bistability [23],[26],[30],[31].

Attainment of negative feedback is not deducible from examining network diagrams such as those in Figure 1, which may bias the observer into assuming that the feedback is necessarily positive. It is ultimately related to bifunctionality of the SHK enzyme that can both increase and decrease the fraction of activated RR. Our results demonstrate a mechanism for creating a negative feedback loop that depends on the existence of a pathway for RR phosphorylation independent of cognate SHK activity. Intuitively, when SHK is the sole source of RR phosphorylation and dephosphorylation the system output is robust and insensitive to SHK concentration [14],[32]. However, when an additional feedback-independent flux of RR phosphorylation exists, upregulation of SHK can disproportionately increase the phosphatase flux resulting in negative feedback. Other mechanisms of attaining negative feedback may exist, but we have failed to identify them (cf. Text S1, Table S2 and Figure S2).

If exogenous phosphorylation is an important mechanism in TCSs, why is it not frequently detected? One proposal is that existence of the phosphatase activity of SHKs results in buffering, or suppression of exogenous phosphorylation and thereby reduces or eliminates crosstalk with other TCSs [33],[34]. As a result, an exogenous phosphorylation flux may not lead to a large effect on the levels of activated regulator and therefore existence of cross-talk may be difficult to detect in wild-type systems [18]. Our modeling predictions support this conclusion: phosphorylated RR can be kept at a low level by phosphatase activity while still maintaining effectively negative feedback enabled by exogenous phosphorylation.

An alternative model for feedback-induced overshoot that does not directly invoke negative feedback is a mechanism for dynamic regulation of SHK kinase or phosphatase activity. We considered two mechanisms: ATP-SHK interactions that alter SHK activities, and a temporal delay in SHK maturation such that the intermediate species has kinase but not phosphatase activity (Text S1). We were unable to produce overshoot kinetics with the ATP-SHK model. The SHK maturation model can produce overshoot, but every case we found requires unrealistic component concentrations and invokes a strong assumption without experimental evidence.

### Relating model predictions to experimental evidence for negative feedback in TCSs

The best characterized system that may be attaining negative feedback is *Salmonella* PhoPQ. The evidence is compelling: it displays overshoot kinetics [12] and increasing SHK expression from an inducible plasmid can reduce expression of a PhoP-regulated gene [2]. However, deleting functional SHK by insertion of a phage-derived element sometimes results in a low level of RR activity [2],[35], in contrast to what would be predicted by the exogenous phosphorylation model in the absence of other differences between the wild-type and mutant.

A likely explanation is that the SHK insertion somewhat destabilizes the transcript in the mutant. In some genes, insertion of stop codons has this effect [36]. Several TCSs also undergo transcript processing with much greater stability of the monocistronic RR mRNA than the polycistronic or SHK-only mRNAs in *E. coli* [37]. If the insertion disrupts mRNA processing, the resulting polycistronic mRNA may have a shorter half-life with resultant lower rate of protein synthesis. The default parameter set for our model predicts that ∼2.5-fold increase in mRNA degradation rate is sufficient for basal expression (Figure S6). Other PhoQ-disabling insertions may not have the same effect on mRNA stability, resulting in overexpression of PhoP in these cases. Indeed, PhoP overexpression resulting from different PhoQ mutagenesis experiments has been observed [38].

Our predictions are consistent with experiments showing overshoot kinetics, which are only attainable with negative feedback (Figure 2). Furthermore, there are multiple established mechanisms for exogenous phosphorylation of RRs [16]. Our model offers an explanation for the attainment of diverse experimental results relating to feedback in TCSs, including overshoot kinetics [12], “learning” effects [3], and feedback effects that only occur for extreme signaling conditions [24]. However, more direct experimental tests are still needed to determine if negative feedback commonly emerges as a characteristic of TCSs in nature.

### Tuning feedback in two-component systems

In some cases, the effective feedback sign in TCSs depends on signal level, and the dynamic characteristics of the system follow the reversal of that sign (Figure 4), suggesting on-the-fly reversal of feedback sign as an adaptive signaling mechanism. Tuning the feedback sign in this way allows a fast response to the initial signal with negative feedback for rapid induction of the new transcriptional program. When the signal is high and persistent, the feedback sign is switched to positive, filtering out transient signaling interruptions and increasing the attainable range of signaling.

We also found some cases where the effective sign of feedback reverses between negative and positive more than once (Figure 4A), being positive at low signal then negative at intermediate signals and eventually positive again at very high signals. A physiologically relevant function for positive feedback at very low signal levels is to create a signal threshold, below which the response is slow, and above which the system responds rapidly to a decisive signal.

The functional consequences of transcriptional feedback are surprisingly flexible for a system with only two interacting proteins. Previous models suggest that TCSs without the crosstalk effect are robust, or insensitive to variations of protein concentrations [14],[32]. The flexibility to tune feedback depending on the signal level appears to be a sacrifice to robustness. Explicit determination of robustness is beyond the scope of this work, but sensitivity to perturbations of one parameter does not necessarily imply that other aspects of robustness are lost. Further, possible evolutionary advantages of flexibility are clear: feedback to both TCS genes in this model enables characteristics of negative and positive feedback that would not be attainable with transcriptional feedback to RR or SHK alone (Figure 5).

Tuning of the feedback sign is reminiscent of other results showing a diverse response without explicitly rewiring the network. Some systems have been shown to transition between graded monostable and discrete bistable steady state dose responses [23],[39],[40]. On evolutionary timescales, evolvable motifs may be capable of adapting to many different functions without disrupting the network architecture [41]. TCSs may be similarly adaptable, but on a short biochemical timescale.

### Conclusions and suggested experiments

We propose a model for TCSs whereby transcriptional feedback shows diverse physiologically relevant effects, including negative feedback that gives rise to fast overshooting responses and positive feedback that better filters transient signal interruptions.

In order to determine the effective feedback sign *in vivo*, we suggest that a direct test is necessary. A conceptually straightforward way to test the feedback sign is to synthetically engineer an open-loop system under an inducible promoter and find the inducer level for which RR and SHK concentration match their wild-type values. Exploring changes in downstream transcriptional activity as a function of inducer concentration would allow direct determination of the feedback sign. (This was done in *E. coli* PhoPQ [24], which shows characteristics of effectively zero feedback at small signals and positive feedback at large signals; c.f. Text S4). A similar experimental set-up with just one protein on an inducible promoter can be used to test feedback synergy and trade-off predictions (Figure 5). Alternatively, TCS point mutations may alter transcriptional or translational efficiency (Reactions 1–2 or 6–7 in Table 1). With a reduction in gene expression efficiency, steady state RRP concentrations will diverge depending on feedback sign (Figure S7 shows predicted effects of such an experiment). Predicted quantitative effects of this method are more pronounced with positive than negative feedback. Thus, the low gains in the negative feedback regime may make steady state effects of the feedback difficult to detect. Methods similar to those used in previous studies exploring crosstalk between TCSs [18] may be useful to determine if *Salmonella* PhoPQ is subject to exogenous phosphorylation.

Many biological networks represent a balance of stimulatory and inhibitory effects. Here we have shown that this balance leads to flexibility and diversity in the functional role of the transcriptional feedback loop. This should guide toward a deeper understanding of how interactions in biological networks may have evolved to allow successful responses to a wide array of conditions.

## Methods

### Mathematical model

We constructed a mathematical model extending previous TCS models [14],[15]. As outlined in the Results section, this model includes reactions for production/degradation, regulation of expression from TCS and downstream genes, and phosphorylation/dephosphorylation of RR by an exogenous source. Included reactions are summarized in Table 1.

### Software and simulations

Models were generated using BioNetGen 2.0.46 [42], and imported for analysis in Mathematica 6.0 with MathSBML 2.7.1-07-Dec-2007 [43]. Monte Carlo parameter sampling was done for parameters varied with log-uniform distributions, having intervals constrained as described in Table S1. Randomly generated parameter sets were tested for physical realizability and consistency with known characteristics of TCSs using the following criteria:

1. mRNA copies <100/cell;
2. mRNA degradation faster than dilution of proteins;
3. basal rate of gene expression lower than feedback-regulated rate: *k*_txnbasal_ < *k*_txn_;
4. sufficient induction of transcriptionally active RR after signaling: [RRP_2_](*k*_ph_ = 10^−3^)−[RRP_2_](*k*_ph_ = 10) >1 molecule/cell.
5. low transcriptional activity of RR when signal is absent: [RRP_2_](*k*_ph_ = 10) and [RRP_2_](*k*_ph_ = 10; *k*_txn_ = 0) ≤1 molecule/cell
6. low downstream gene expression when signal is absent: [DS protein](*k*_ph_ = 10) and [DS protein](*k*_ph_ = 10; *k*_txn_ = 0) differ by less than 10%
7. Sufficient overall elevation of components after activation signaling: [DS], [RRP_2_] and [RR_tot_] change more than 2-fold between *k*_ph_ = 10 and *k*_ph_ = 0.1.

Response times were calculated as elevation time for attaining either 50% (τ_50_) or 95% (τ_95_) of the activated steady state. The former is reported for responses where post-transcriptional kinetics dominate a response while the latter is reported when transcriptional induction dominated response times. In practice, qualitative differences between these measures are minimal.

Two sample parameter sets were selected to have similar open-loop induction kinetics for the purposes of illustrating differences in responses between positive and negative feedback (Figure 3). Negative feedback example parameters: *k_ap_* = 0.1706, *k_ad_* = 9.786, *k_pt_* = 0.2028, *k_tp_* = 0.1368, *k_b_* = 2.314, *k_d_* = 0.9237, *k_b1_* = 6.261, *k_d1_* = 0.001825, *k_RRPdm_* = 9.794, *k_RRPmd_* = 0.1411, *k_txn_* = 2.115×10^−5^, *k_SKtsn_* = 0.04708, tsn mult = 5.616, *k_txnbasal_* = 2.245×10^−6^, *K_mDS_* = 0.01224, *K_m_* = 0.004298, *k_mRNAdeg_* = 0.001383, *k_exp_* = 0.02668, *K_mexp_* = 0.1361, *k_exd_* = 4.218×10^−5^, *K_mexd_* = 1.388. Positive feedback example parameters: *k_ap_* = 8.408, *k_ad_* = 0.004832, *k_pt_* = 1.392, *k_tp_* = 0.05183, *k_b_* = 0.07871, *k_d_* = 0.001172, *k_b1_* = 2.395, *k_d1_* = 0.002223, *k_RRPdm_* = 2.665, *k_RRPmd_* = 7.421, *k_txn_*  = 0.0006604, *k_SKtsn_* = 0.005240, tsn mult = 6.190, *k_txnbasal_* = 3.413×10^−5^, *K_mDS_* = 0.06276, *K_m_* = 0.003958, *k_mRNAdeg_* = 0.007446, *k_exp_* = 3.452×10^−6^, *K_mexp_* = 0.0004101, *k_exd_* = 5.017×10^−6^, *K_mexd_* = 0.0001661.

### Determination of effective feedback sign, overshoot kinetics and induction range

We used Monte Carlo sampling of parameter values to numerically determine the range of realizable dynamic behaviors in the autoregulated TCSs focusing on the sign of autoregulation and possibility of overshoot behavior. Each parameter set was used to simulate activation kinetics after a signal at *t* = 0 s by changing phosphatase activity (*k*_ph_) from a high, resting state (10 s^−1^) to a low level (1 s^−1^). To determine the effect of feedback, we used an open-loop version of the model in which an exogenous regulator R_0_ rather than (RRP)_2_ controls SK/RRP production. The effective sign of feedback is determined by the sign of the gain defined as the derivative 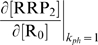. If this gain is positive (
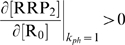
) the transcriptional feedback is positive; if the gain is negative (
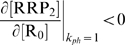
), the feedback is negative. A subset of cases predict RRP_2_ overshoot without mRNA overshoot. This phenomenon is not dependent on transcriptional feedback and is not of interest here. Therefore we do not analyze these parameter sets.

Induction range (Figure 5) is defined as the difference between RRP_2_ at high and low signal limits, normalized to the case without feedback: 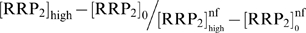.

### Genetic algorithm

To determine the range of behaviors attainable by the model, we evolved examples conforming to specific criteria using a simple genetic algorithm. Using a set of seed parameter sets, the algorithm randomly perturbs parameters and selects sets with the highest value for a fitness function. This function depends on the desired criteria.

To select a parameter set that conforms to known important characteristics of *Salmonella* PhoPQ after a signal, the desired characteristics include at least 2-fold induction of (1) [RRP_2_] over the resting level (*f*_1_); (2) RR_tot_ over the resting level (*f*_2_); and (3) peak RRP_2_ concentration over activated steady state RRP_2_ (*f*_3_). The following fitness function selects for these criteria, with each criterion noted: 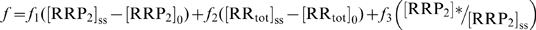 where 
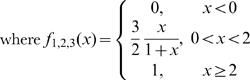
.

## Supporting Information

Text S1 Alternative models of two-component system kinetics
(0.07 MB PDF)

Text S2 Partitioned Monte Carlo sampling in a simplified model
(0.03 MB PDF)

Text S3 Non-steady state open loop gains and response times
(0.03 MB PDF)

Text S4 Relating overshoot kinetics to steady state response
(0.03 MB PDF)

Figure S1 Characteristics of two-component system model calibrated to resemble Salmonella PhoPQ. Dynamics of RRP2 (A), total RR (B), total SHK (C), and a downstream protein upregulated by RRP2 (D).
(0.08 MB PDF)

Figure S2 Schematic of a two-component system model with sensor histidine kinase (SHK) maturation. This model is identical to the main text, except that SHK-independent phosphorylation of response regulator (RR) is disallowed, and newly produced SHK enters a temporary state where it is capable of binding and phosphorylating RR, but cannot catalyze the phosphatase reaction. Newly translated SHK_0 matures at a rate kconf.
(0.05 MB PDF)

Figure S3 Sampling of partitioned parameter space in a simplified two-component system model. (A) The model was simplified by eliminating as many variables as possible while retaining the capability for negative open-loop gain. (B) Distribution of negative and positive open-loop gain cases for fraction of exogenous phosphorylation flux JE/(JE + JS). Histogram bins containing more than 105 members were cut off for clarity. (C) Distribution of cases with feedback-induced overshoot >10% over the activated steady state.
(0.08 MB PDF)

Figure S4 Two-component system kinetics with non-steady state open loop gain that switches between positive and negative.
(0.09 MB PDF)

Figure S5 Relationship between steady state dose-response and overshoot kinetics in two-component systems.
(0.15 MB PDF)

Figure S6 Altered mRNA stability in a simulated SHK knockout changes total RR concentrations. Wildtype concentrations at the default degradation rate for various signal levels are shown for reference. All simulations use the default parameter set (Table S1).
(0.05 MB PDF)

Figure S7 Predicted steady-state effects of perturbing translational efficiency.
(0.04 MB PDF)

Table S1 Intervals for Monte Carlo sampling and reference parameter set
(0.02 MB PDF)

Table S2 Reaction mechanisms for a generalized two-component system model
(0.02 MB PDF)
