# Supplementary material for: Adaptable Functionality of Transcriptional Feedback in Bacterial Two-Component Systems
Source: PLoS Comput Biol. 2010 Feb 12;6(2):e1000676. doi: 10.1371/journal.pcbi.1000676 (PMC2820520; doi:10.1371/journal.pcbi.1000676)
Supplement: Text S1 — Alternative models of two-component system kinetics (0.07 MB PDF) [file pcbi.1000676.s001.pdf]

Supplemental Text for: Adaptable Functionality of Transcriptional Feedback in Bacterial Two-Component Systems by J. Christian J. Ray and Oleg A. Igoshin

Text S1: Alternative models of two-component system kinetics

The results presented in the main text correspond to a model in which some source of exogenous phosphorylation and/or dephosphorylation elicits effectively negative transcriptional feedback in a two-component system. An alternative possibility is a mechanism for dynamic regulation of kinase or phosphatase activity of the sensor histidine kinase (SHK). Here we describe two alternate mechanisms for modulating the balance of kinase and phosphatase activity. One is a generalized kinetic scheme that allows ATP and ADP interactions with SHK and response regulator (RR), along with other generalizations. The second is a model where maturation of SHK is delayed so that it transiently has kinase, but not phosphatase, activity. The goal was to attain overshoot in a feedback-dependent manner with them. In the first model, we were unable to do so. The second is a strong assumption, and apparently requires unrealistic molecular concentrations for overshoot kinetics. We explore the models in more detail below.

<u>ATP interaction mechanism</u>

In this model, we considered a very general set of interactions, including every possible regulatory step by SHK-ATP and SHK-ADP interactions, and excluding an exogenous mechanism for phohphorylating response regulator (Table S2). Since SHK activity is modulated by a conformational switch, we included two conformations for each form of SHK: one with full phosphatase activity, and one lacking phosphatase activity. The effective phosphatase rate emerges from the concentrations of the two conformations of RRP.SHK: $k_{ph}^{eff} = \frac{k_{ph}^{max}[\text{RRP.SHK}']}{[\text{RRP.SHK}']+[\text{RRP.SHK}]}$ where SHK has no phosphatase activity and SHK′ has full phosphotase activity. In practice, if the conformational switch is sufficiently

fast a single conformation suffices with $k_{ph} = k_{ph}^{eff}$, as in the main text. The resulting model is in other respects the same as Figure 1, with transcriptional production and degradation.

We subjected this model to extensive Monte Carlo sampling and simulations with a genetic algorithm assuming a minimal fitness function (Methods), selecting only for an overshoot response. We placed no specific constraints on parameter values, allowing possible submodels to arise out of this generalized model. We were unable to produce an overshoot with this model or any submodels from it.

Delayed sensor histidine kinase maturation mechanism

Another mechanism for dynamic regulation of kinase and phosphatase activity is for the SHK to undergo a temporary state where it has kinase activity, but no phosphatase activity (Figure S2). Then, as the system reaches the activated state, the fraction of SHK in the immature state would be decreased, increasing the overall phosphatase activity and reducing RRP concentrations to below peak levels. Aside from an exogenous RR phosphorylation mechanism, this model is otherwise identical to that of the main text.

Using a genetic algorithm, we found cases where this model is capable of producing an overshoot. However, these cases all had unrealistically high concentrations of SHK and RR. Using stricter fitness criteria, we were unable to isolate a case with overshoot. Even if such a case exists and we were unable to find it, this model is not preferable to the exogenous phosphorylation model presented in the main text. The delayed maturation model requires a strong, counterintuitive assumption—namely, that somehow SHK kinase activity precedes phosphatase activity for some SHK molecules. This has not been observed in any TCS to our knowledge.
