# Supplementary material for: Adaptable Functionality of Transcriptional Feedback in Bacterial Two-Component Systems
Source: PLoS Comput Biol. 2010 Feb 12;6(2):e1000676. doi: 10.1371/journal.pcbi.1000676 (PMC2820520; doi:10.1371/journal.pcbi.1000676)
Supplement: Text S2 — Partitioned Monte Carlo sampling in a simplified model (0.03 MB PDF) [file pcbi.1000676.s002.pdf]

Supplemental Text for: Adaptable Functionality of Transcriptional Feedback in Bacterial Two-Component Systems by J. Christian J. Ray and Oleg A. Igoshin

Text S2: Partitioned Monte Carlo sampling in a simplified model

Monte Carlo sampling in the main text explores a 21-dimensional parameter space, making extensive coverage of the space impractical. To more extensively explore the parameter space and further test our conclusions that (i) overshoot occurs with negative open-loop gain or (ii) negative open-loop gain requires exogenous phosphorylation, we simplified the model. Any reactions not strictly necessary to demonstrate overshoot and negative open-loop gain were removed. The resulting simplified model has 13 parameters in the sample space (Figure S3A). We partitioned each parameter into 4 intervals, and sampled each once. This resulted in a sampling of 67,108,864 discrete regions of parameter space. We discarded cases that cause numerical computation errors . Figure S3B shows the distribution of exogenous phosphorylation ratios $J_E/(J_E + J_S)$ (fractions of RR phosphorylation not from cognate SHK) to have a lower bound of approximately 10^{-4} for effectively negative feedback[1] (open-loop gain $\left.\frac{\partial[\mathrm{RRP_2}]}{\partial[\mathrm{R_0}]}\right|_{k_{ph}=0.1} < -0.01$) but not for positive ($\left.\frac{\partial[\mathrm{RRP_2}]}{\partial[\mathrm{R_0}]}\right|_{k_{ph}=0.1} > 0.01$) . All cases found to exhibit feedback-dependent overshoot have effectively negative feedback (Figure S3C). Interestingly, the model frequently predicted significant overshoot for low negative open-loop gains. We conclude that extensive parameter sampling supports our conclusions.

[1] For clarity we only focused on the cases with absolute value of open loop-gains larger than 1% to discard the large number of cases with effectively zero open-loop gain.
