# Supplementary material for: Adaptable Functionality of Transcriptional Feedback in Bacterial Two-Component Systems
Source: PLoS Comput Biol. 2010 Feb 12;6(2):e1000676. doi: 10.1371/journal.pcbi.1000676 (PMC2820520; doi:10.1371/journal.pcbi.1000676)
Supplement: Text S3 — Non-steady state open loop gains and response times (0.03 MB PDF) [file pcbi.1000676.s003.pdf]

Supplemental Text for: Adaptable Functionality of Transcriptional Feedback in Bacterial Two-Component Systems by J. Christian J. Ray and Oleg A. Igoshin

Text S3: Non-steady state open loop gains and response times

Monte Carlo parameter sampling shows that, in most cases, negative feedback speeds induction times copared to positive feedback (Figure 3). A small subset of negative feedback cases appears to contradict this rule. The measure of feedback sign used is the steady state open loop gain, $\left.\frac{\partial[\mathrm{RRP_2}]}{\partial[\mathrm{R_0}]}\right|_{k_{ph}=0.1}$. However, after an instantaneous change of the parameter k_{ph} indicating the signal, the system relaxes from a non-steady state point. Assuming that biochemical kinetics are sufficiently fast, feedback-regulated induction dynamics are set by the transcriptional timescale, and intermediate open loop gains can be approximated by quasi-steady state points $\left.\frac{\partial[\mathrm{RRP_2}]}{\partial[\mathrm{R_0}=\mathrm{RRP_2}(t)]}\right|_{k_{ph}=0.1}$ for time points t preceding the activated steady state. In cases where negative steady state open loop gain apparently slows the system response, the non-steady state gains are positive for much of the induction time, converging to a negative gain at a late timepoint. We thus found some instances of TCS induction kinetics that have negative steady state gain, but with kinetics dominated by positive non-steady state gain (Figure S4). As noted in the main text, these cases do not exhibit overshoot, which contributes significantly to speeding responses.
