# Supplementary material for: Adaptable Functionality of Transcriptional Feedback in Bacterial Two-Component Systems
Source: PLoS Comput Biol. 2010 Feb 12;6(2):e1000676. doi: 10.1371/journal.pcbi.1000676 (PMC2820520; doi:10.1371/journal.pcbi.1000676)
Supplement: Text S4 — Relating overshoot kinetics to steady state response (0.03 MB PDF) [file pcbi.1000676.s004.pdf]

Supplemental Text for: Adaptable Functionality of Transcriptional Feedback in Bacterial Two-Component Systems by J. Christian J. Ray and Oleg A. Igoshin

Text S4: Relating overshoot kinetics to steady state response

In recent work with *E. coli* PhoPQ, Miyashiro and Goulian [1] compared the steady state dose response between the wild-type case with feedback, and a case lacking the feedback-regulated promoter. They found that the dose response is nearly identical for many signal levels, but varies greatly at high levels of signal. This raises the question of what, if any, dynamic effects can occur without significant changes in feedback. We tested the hypothesis that there is a quantitative upper limit to the overshoot level as a function of the steady state. To calculate this comparison, we took steady state [RRP_{tot}] in the wild-type case and an open-loop case with basal TCS production, restricted to the range between inactive and active k_{ph} values (i.e. $k_{ph} \in [0.1, 10]$). Finding $\max(|\Delta_{ss}|)/[RRP_{tot}]$ and comparing to Δ_{os}^{norm}, most cases from the Monte Carlo sample had $\Delta_{os}^{norm} < \max(|\Delta_{ss}|)/[RRP_{tot}]$ (Figure S5A). This is consistent with the hypothesis that *E. coli* PhoPQ does not exhibit a large overshoot at lower signal strengths.

One case predicted a violation of this rule. To understand this case further, we varied a single parameter to find the transition point $\Delta_{os}^{norm} = \max(|\Delta_{ss}|)/[RRP_{tot}]$. This case showed two regions of variation in the signal interval $k_{ph} \in [0.1, 10]$ (Figure S5). When point *a* in Figure S5D was sufficiently large, the rule was violated. This appears to a consistent effect regardless of the parameter varied to alter the overshoot effect. Thus, a difference between steady state RRP_{tot} for intermediate activation levels (here, $0.1 < k_{ph} < 10$) may contribute to the level of overshoot.

Text S4 Reference

1. Miyashiro T, Goulian M (2008) High Stimulus Unmasks Positive Feedback in an Autoregulated Bacterial Signaling Circuit. Proc Natl Acad Sci U S A 105: 17457-17462.
