# Supplementary material for: Adaptable Functionality of Transcriptional Feedback in Bacterial Two-Component Systems
Source: PLoS Comput Biol. 2010 Feb 12;6(2):e1000676. doi: 10.1371/journal.pcbi.1000676 (PMC2820520; doi:10.1371/journal.pcbi.1000676)
Supplement: Figure S1 — Characteristics of two-component system model calibrated to resemble Salmonella PhoPQ. Dynamics of RRP2 (A), total RR (B), total SHK (C), and a downstream protein upregulated by RRP2 (D). (0.08 MB PDF) [file pcbi.1000676.s005.pdf]

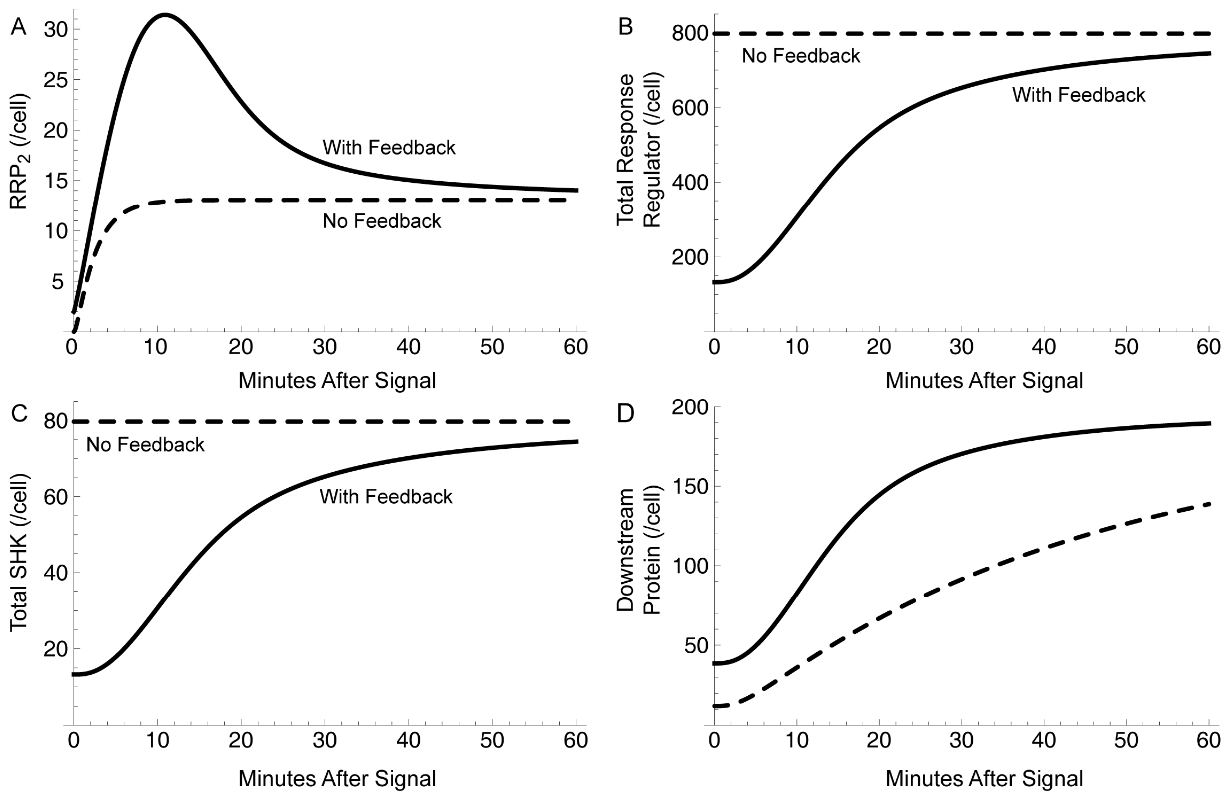

Figure S1. Characteristics of two-component system model calibrated to resemble *Salmonella* PhoPQ. Dynamics of RRP_2 (A), total RR (B), total SHK (C), and a downstream protein upregulated by RRP_2 (D).
