# Supplementary material for: Adaptable Functionality of Transcriptional Feedback in Bacterial Two-Component Systems
Source: PLoS Comput Biol. 2010 Feb 12;6(2):e1000676. doi: 10.1371/journal.pcbi.1000676 (PMC2820520; doi:10.1371/journal.pcbi.1000676)
Supplement: Figure S2 — Schematic of a two-component system model with sensor histidine kinase (SHK) maturation. This model is identical to the main text, except that SHK-independent phosphorylation of response regulator (RR) is disallowed, and newly produced SHK enters a temporary state where it is capable of binding and phosphorylating RR, but cannot catalyze the phosphatase reaction. Newly translated SHK_0 matures at a rate kconf. (0.05 MB PDF) [file pcbi.1000676.s006.pdf]

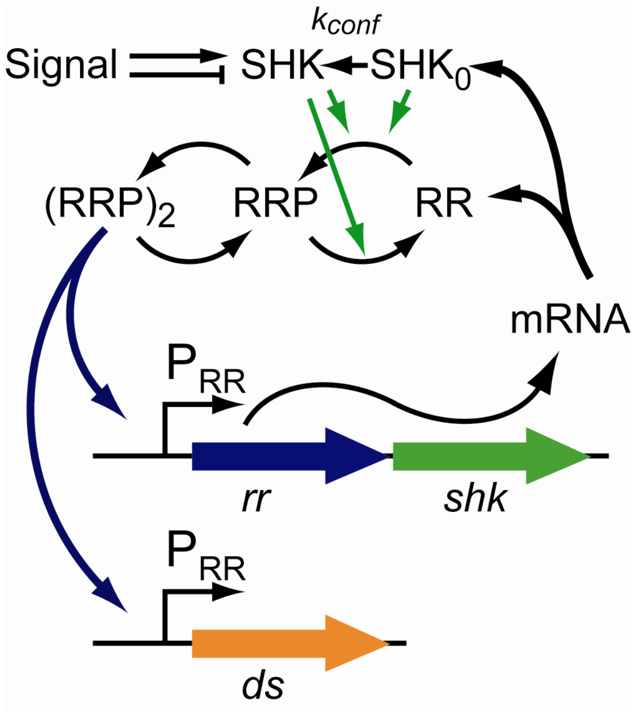

Figure S2. Schematic of a two-component system model with sensor histidine kinase (SHK) maturation. This model is identical to the main text, except that SHK-independent phosphorylation of response regulator (RR) is disallowed, and newly produced SHK enters a temporary state where it is capable of binding and phosphorylating RR, but cannot catalyze the phosphatase reaction. Newly translated SHK_0 matures at a rate k_{conf}.
