# Supplementary material for: Adaptable Functionality of Transcriptional Feedback in Bacterial Two-Component Systems
Source: PLoS Comput Biol. 2010 Feb 12;6(2):e1000676. doi: 10.1371/journal.pcbi.1000676 (PMC2820520; doi:10.1371/journal.pcbi.1000676)
Supplement: Figure S3 — Sampling of partitioned parameter space in a simplified two-component system model. (A) The model was simplified by eliminating as many variables as possible while retaining the capability for negative open-loop gain. (B) Distribution of negative and positive open-loop gain cases for fraction of exogenous phosphorylation flux JE/(JE + JS). Histogram bins containing more than 105 members were cut off for clarity. (C) Distribution of cases with feedback-induced overshoot >10% over the activated steady state. (0.08 MB PDF) [file pcbi.1000676.s007.pdf]

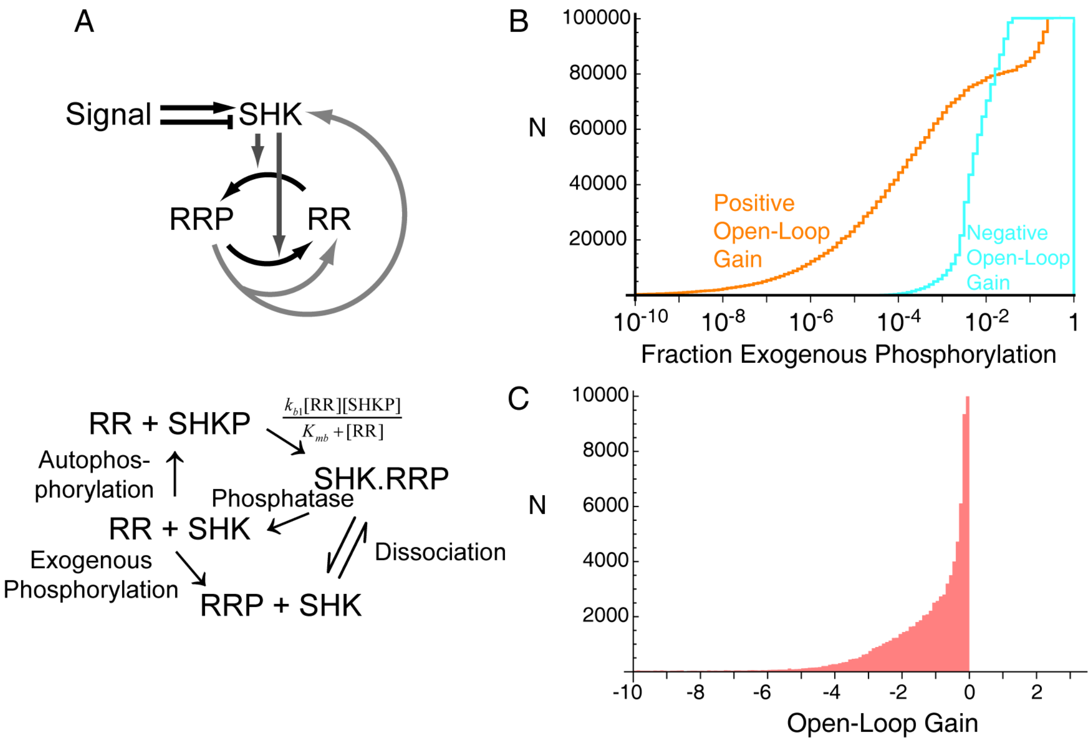

Figure S3. Sampling of partitioned parameter space in a simplified two-component system model. (A) The model was simplified by eliminating as many variables as possible while retaining the capability for negative open-loop gain. (B) Distribution of negative and positive open-loop gain cases for fraction of exogenous phosphorylation flux $J_E/(J_E + J_S)$. Histogram bins containing more than 10^5 members were cut off for clarity. (C) Distribution of cases with feedback-induced overshoot > 10% over the activated steady state. Intervals for parameter sampling were the same as Table S1 with the following exceptions: $k'_{txn} \in [4.33\times10^{-5}, 100.]$; $k'_{txnbasal} \in [4.33\times10^{-6}, 1.0]$; $K_{mb} \in [10^{-4}, 10]$. $k'_{txnbasal}$ and k'_{txn} are derived from dividing $k_{txnbasal}$ and k_{txn} by $k_{mRNAdeg}$. Production rates of RR and SHK are $k_{tsn}\left(\frac{k'_{txn}[\mathrm{RRP}]^2}{K_{mtxn}^2+[\mathrm{RRP}]^2}+k'_{txnbasal}\right)$ for $k_{tsn} = k_{tsnRR}$, k_{tsnSHK}, respectively.
