# Supplementary material for: Adaptable Functionality of Transcriptional Feedback in Bacterial Two-Component Systems
Source: PLoS Comput Biol. 2010 Feb 12;6(2):e1000676. doi: 10.1371/journal.pcbi.1000676 (PMC2820520; doi:10.1371/journal.pcbi.1000676)
Supplement: Figure S4 — Two-component system kinetics with non-steady state open loop gain that switches between positive and negative. (0.09 MB PDF) [file pcbi.1000676.s008.pdf]

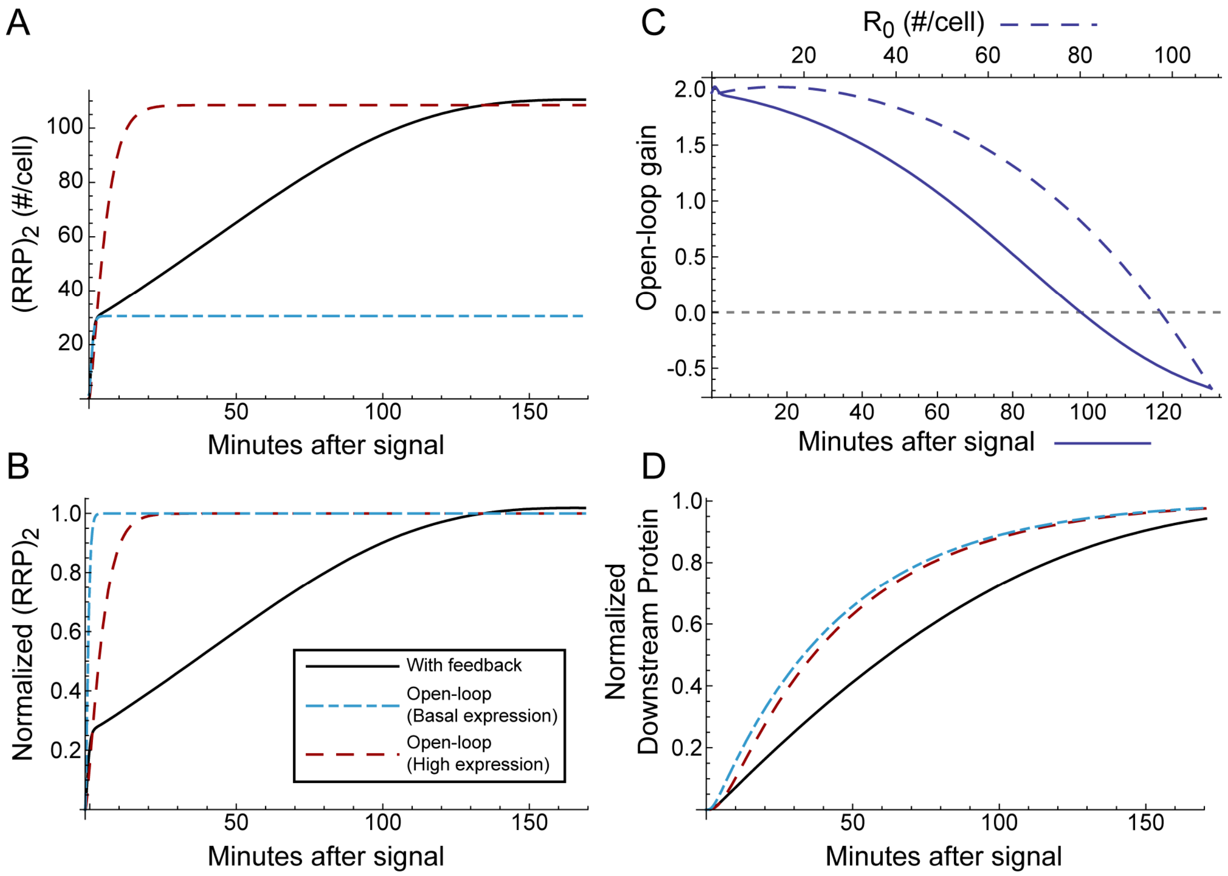

Figure S4. Two-component system kinetics with non-steady state open loop gain that switches between positive and negative. A-B. Induction of transcriptionally active response regulator dimer $(RRP)_2$ in a case with negative steady state open loop gain and open loop dynamics with basal ($R_0 = (RRP)_2 |_{k_{ph}=10}$) or high expression ($R_0 = (RRP)_2 |_{k_{ph}=0.1}$). C. Non-steady state open loop gain switches between positive and negative over time. D. Downstream protein induction is slowed by feedback in this case. Parameters: k_{ap}=0.185045, k_{ad}=5.16274, k_{pt}=3.04614, k_{tp}=2.3304, k_b=4.06472, k_d=0.0528448, k_{b1}=0.338406, k_{d1}=0.04235, k_{RRPdm}=0.962574, k_{RRPmd}=1.43538, k_{txn} =0.0000755422, k_{SKtsn}=0.036068, tsn mult=15.4986, $k_{txnbasal}$ =3.41719×10^{-6}, K_{mDS}=0.15259, K_m =1.40665, $k_{mRNAdeg}$=0.0115463, k_{exp}=0.00995825, K_{mexp}=0.216291, k_{exd}=8.23057×10^{-6}, K_{mexd}=0.0282159.
