# Supplementary material for: Adaptable Functionality of Transcriptional Feedback in Bacterial Two-Component Systems
Source: PLoS Comput Biol. 2010 Feb 12;6(2):e1000676. doi: 10.1371/journal.pcbi.1000676 (PMC2820520; doi:10.1371/journal.pcbi.1000676)
Supplement: Figure S5 — Relationship between steady state dose-response and overshoot kinetics in two-component systems. (0.15 MB PDF) [file pcbi.1000676.s009.pdf]

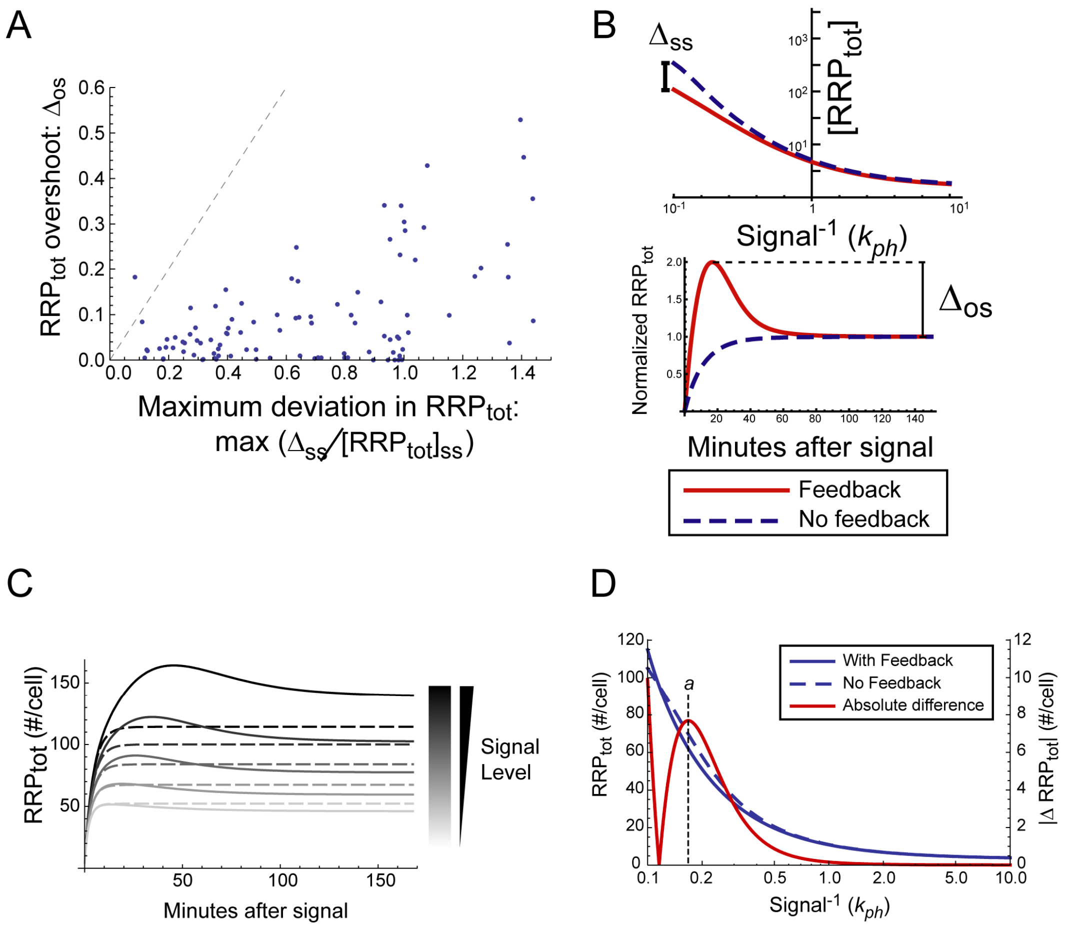

Figure S5. Relationship between steady state dose-response and overshoot kinetics in two-component systems. A. Monte Carlo sampling suggests a limit to overshoot level (Δ_{os}) related to the maximum deviation in total phosphoylated response regulator between the system with feedback and the equivalent open-loop system with basal gene expression levels: $\max(\Delta_{ss}/[RRP_{tot}]_{ss})$. B. Illustration of the relationship between Δ_{os} and Δ_{ss}. C-D. Violation of this rule may arise if intermediate steady state differences contribute to overshoot. Parameters for the case violating the rule: k_{ap}=0.11218, k_{ad}=2.1113, k_{pt}=0.42707, k_{tp}=1.6747, k_b=6.6956, k_d=1.8197, k_{b1}=0.011620, k_{d1}=0.0041530, k_{RRPdm}=3.1596, k_{RRPmd}=2.0857, k_{txn} =0.000014292, k_{SKtsn}=0.038183, tsn mult=13.463, $k_{txnbasal}$=4.0570×10^{-6}, K_{mDS}=0.0040868, K_m=0.0063422, $k_{mRNAdeg}$=0.015268, k_{exp}=0.0025826, K_{mexp}=0.64509, k_{exd}=0.000044020, K_{mexd}=1.56357.
