# Supplementary material for: Adaptable Functionality of Transcriptional Feedback in Bacterial Two-Component Systems
Source: PLoS Comput Biol. 2010 Feb 12;6(2):e1000676. doi: 10.1371/journal.pcbi.1000676 (PMC2820520; doi:10.1371/journal.pcbi.1000676)
Supplement: Figure S6 — Altered mRNA stability in a simulated SHK knockout changes total RR concentrations. Wildtype concentrations at the default degradation rate for various signal levels are shown for reference. All simulations use the default parameter set (Table S1). (0.05 MB PDF) [file pcbi.1000676.s010.pdf]

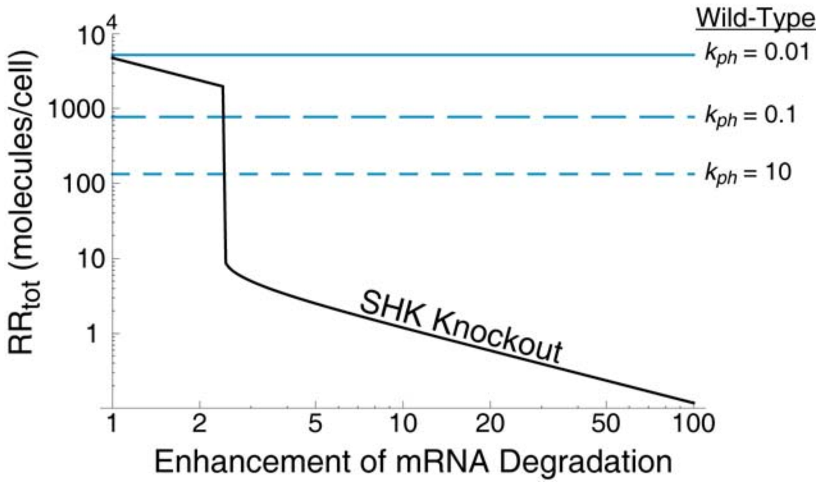

Figure S6. Altered mRNA stability in a simulated SHK knockout changes total RR concentrations. Wild-type concentrations at the default degradation rate for various signal levels are shown for reference. All simulations use the default parameter set (Table S1).
