# Supplementary material for: Adaptable Functionality of Transcriptional Feedback in Bacterial Two-Component Systems
Source: PLoS Comput Biol. 2010 Feb 12;6(2):e1000676. doi: 10.1371/journal.pcbi.1000676 (PMC2820520; doi:10.1371/journal.pcbi.1000676)
Supplement: Figure S7 — Predicted steady-state effects of perturbing translational efficiency. (0.04 MB PDF) [file pcbi.1000676.s011.pdf]

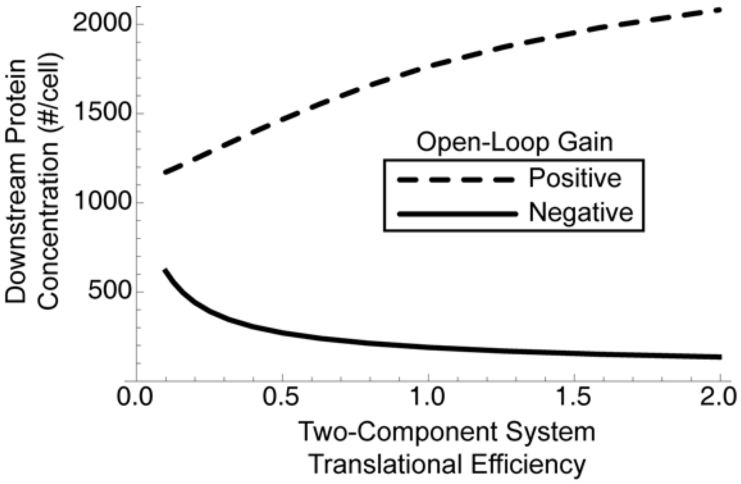

Figure S7. Predicted steady-state effects of perturbing translational efficiency. Rates of response regulator and sensor histidine kinase translation (k_{RRtsn}, k_{SHKtsn}) were changed by an efficiency multiplier using the reference parameter set at k_{ph} = 0.1 (negative gain) and k_{ph} = 0.001 (positive gain). A positively regulated downstream protein readout was taken one hour after signal.
