# Supplementary material for: Adaptable Functionality of Transcriptional Feedback in Bacterial Two-Component Systems
Source: PLoS Comput Biol. 2010 Feb 12;6(2):e1000676. doi: 10.1371/journal.pcbi.1000676 (PMC2820520; doi:10.1371/journal.pcbi.1000676)
Supplement: Table S1 — Intervals for Monte Carlo sampling and reference parameter set (0.02 MB PDF) [file pcbi.1000676.s012.pdf]

Table S1. Intervals for Monte Carlo sampling and reference parameter set

Parameter	Min	Max	Median –[a]	Median +[b]	Reference[c]	Units
k_{ap}	0.1	10.0	0.3820	1.046	3.0	s^{-1}
k_{ad}	0.001	10.0	0.1376	0.08304	0.04	s^{-1}
k_{pt}	0.1	10.0	1.232	1.209	3.0	s^{-1}
k_{tp}	0.001	10.0	0.1138	0.05781	2.0	s^{-1}
k_b	0.01	10.0	4.268	0.4657	5.0	$\mu M^{-1}s^{-1}$
k_d	0.001	10.0	0.09554	0.4155	0.01	s^{-1}
k_{b1}	0.01	10.0	0.7098	0.6791	5.0	$\mu M^{-1}s^{-1}$
k_{d1}	0.001	10.0	0.05328	0.05811	0.01	s^{-1}
k_{RRPdm}	0.1	10.0	1.398	0.7684	1.2	s^{-1}
k_{RRPmd}	0.1	10.0	0.6841	1.401	3.2	$\mu M^{-1}s^{-1}$
k_{txn}	10^{-6}	0.1	0.0002785	0.0001209	0.0007	$\mu M\ s^{-1}$
k_{SHKtsn}	10^{-6}	0.1	0.004151	0.005813	0.015	s^{-1}
RR tsn mult[d]	1.	500.	14.41	13.38	10	-
$k_{txnbasal}$	10^{-7}	0.001	3.335×10^{-6}	3.827×10^{-6}	10^{-6}	$\mu M\ s^{-1}$
K_{mDS}	0.0001	33.2116	0.01454	0.01436	1.0	μM
K_{mtxn}	0.0001	33.2116	0.1454	0.04775	0.22	μM
$k_{mRNAdeg}$	9.99×10^{-4}	0.0231	0.005402	0.005271	0.02	s^{-1}
k_{exp}	10^{-6}	0.1	0.002616	0.00002368	0.001	$\mu M\ s^{-1}$
K_{mexp}	0.0001	10.0	0.01030	0.05857	0.0001	μM
k_{exd}	10^{-6}	0.1	0.00004728	0.0001149	0.0017	$\mu M\ s^{-1}$
K_{mexd}	0.0001	10.0	0.02833	0.07985	0.9	μM

[a]Median value from samples exhibiting negative open-loop gain at k_{ph} = 0.1.

[b]Median value from samples exhibiting positive open-loop gain at k_{ph} = 0.1.

[c]Parameter set chosen to resemble *Salmonella* PhoPQ

[d] $k_{RRtsn} = (\text{RR tsn mult})\, k_{SKtsn}$
