# Supplementary material for: Adaptable Functionality of Transcriptional Feedback in Bacterial Two-Component Systems
Source: PLoS Comput Biol. 2010 Feb 12;6(2):e1000676. doi: 10.1371/journal.pcbi.1000676 (PMC2820520; doi:10.1371/journal.pcbi.1000676)
Supplement: Table S2 — Reaction mechanisms for a generalized two-component system model (0.02 MB PDF) [file pcbi.1000676.s013.pdf]

Table S2. Reaction mechanisms for a generalized two-component system model

Reaction[a]	Forward Rate Constant	Reverse Rate Constant
$SHK_T \rightleftharpoons SHK_D$	k_{td}	k_{dt}
$SHK_D \rightleftharpoons SHK_0$	k_{d0}	k_{0d}
$SHK_T \rightleftharpoons SHK_0$	k_{t0}	k_{0t}
$SHK_T \rightleftharpoons SHK_DP$	k_{ap}	k_{ad}
$SHK_DP.RR \rightleftharpoons SHK_D.RRP$	k_{pt}	k_{tp}
$SHK_TP.RR \rightleftharpoons SHK_T.RRP$	k_{pt}	k_{tp}
$SHK_T.RRP \rightleftharpoons SHK_D.RRP$	k_{tdc}	k_{dtc}
$RRP + SHK_D \rightleftharpoons SHK_D.RRP$	k_b	k_d
$RRP + SHK_T \rightleftharpoons SHK_T.RRP$	k_b	k_d
$RR + SHK_DP \rightleftharpoons SHK_DP.RR$	k_{b1}	k_{d1}
$RRP + SHK_DP \rightleftharpoons SHK_DP.RRP$	k_{b2}	k_{d2}
$RR + SHK_TP \rightleftharpoons SHK_TP.RR$	k_{b1}	k_{d1}
$RRP + SHK_TP \rightleftharpoons SHK_TP.RRP$	k_{b2}	k_{d2}
$RR + SHK_D \rightleftharpoons SHK_D.RR$	k_{b3}	k_{d3}
$RR + SHK_T \rightleftharpoons SHK_T.RR$	k_{b3}	k_{d3}
$SHK_T.RRP \longrightarrow SHK_T.RR$	k_{ph}	-
$SHK_D.RRP \longrightarrow SHK_D.RR$	$\alpha_1\ k_{ph}$	-
$SHK_DP.RRP \longrightarrow SHK_DP.RR$	$\alpha_2\ k_{ph}$	-
$SHK_TP.RRP \longrightarrow SHK_TP.RR$	$\alpha_3\ k_{ph}$	-

[a]All reaction mechanisms in this table occur by mass action. Each SHK species can be in one of two conformations. The last 4 reactions only occur in the phosphatase-active conformation. Translation of SHK mRNA produces SHK_0. All other reactions are the same as the main text.
